# *N*-(2-Iodo­phen­yl)benzene­carbox­imid­amide

**DOI:** 10.1107/S160053681200596X

**Published:** 2012-02-17

**Authors:** Yin-jun Zhang, Dong Wang, Hai-Liang Zhang, Yu-Guang Wang

**Affiliations:** a College of Biological and Environmental Engineering, Zhejiang University of Technology, Hangzhou 310014, People’s Republic of China; b Zhejiang SiXian Pharmaceutical Co. Ltd, ShaoXing 312065, People’s Republic of China

## Abstract

The title compound, C_13_H_11_IN_2_, crystallizes with two independent molecules (*A* and *B*) in the asymmetric unit. The two aromatic rings are inclined to one another by 73.3 (2)° in molecule *A*, and by 74.4 (1)° in molecule *B*. In molecule *A*, the iodophenyl and the phenyl rings are inlclined to the N=C—N plane by 88.0 (4) and 19.0 (4)°, respectively. In molecule *B* the corresponding angles are 85.0 (4) and 20.7 (4)°, respectively. In the crystal, the two molecules are not parallel but have a dihedral angle between the iodophenyl rings of 8.6 (1)°, and 44.5 (2)° between the phenyl rings. The *A* and *B* molecules are linked v*via* N—H⋯N hydrogen bonds to form –*A*–*B*–*A*–*B*– chains propagating along direction [100].

## Related literature

For the application of amidines in the synthesis of heterocyclic compounds, see: Attanasi *et al.* (2010 ▶); Bhosale *et al.* (2010 ▶); Deng & Mani (2010 ▶); Wang *et al.* (2011 ▶); Ohta *et al.* (2010 ▶). For details of the synthetic procedure to yield the title compound, see: Ma *et al.* (2011 ▶); Cortes-Salva *et al.* (2011 ▶).
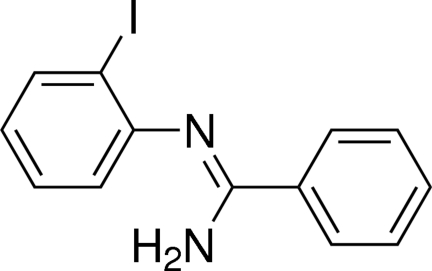


## Experimental

### 

#### Crystal data

C_13_H_11_IN_2_
*M* *_r_* = 322.14
Triclinic, 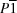

*a* = 10.411 (3) Å
*b* = 11.024 (3) Å
*c* = 11.534 (3) Å
α = 95.501 (3)°
β = 95.065 (3)°
γ = 102.986 (3)°
*V* = 1275.7 (6) Å^3^
*Z* = 4
Mo *K*α radiation
μ = 2.49 mm^−1^
*T* = 296 K
0.49 × 0.44 × 0.30 mm

#### Data collection

CCD area-detector diffractometer
Absorption correction: multi-scan (*SADABS*; Sheldrick, 1996 ▶) *T*_min_ = 0.376, *T*_max_ = 0.523
9707 measured reflections
4716 independent reflections
4076 reflections with *I* > 2σ(*I*)
*R*_int_ = 0.013

#### Refinement

*R*[*F* ^2^ > 2σ(*F* ^2^)] = 0.033
*wR*(*F* ^2^) = 0.079
*S* = 1.04
4716 reflections
289 parameters
H-atom parameters constrained
Δρ_max_ = 0.94 e Å^−3^
Δρ_min_ = −1.34 e Å^−3^

### 

Data collection: *SMART* (Bruker, 2004 ▶); cell refinement: *SAINT* (Bruker, 2004 ▶); data reduction: *SAINT*; program(s) used to solve structure: *SHELXS97* (Sheldrick, 2008 ▶); program(s) used to refine structure: *SHELXL97* (Sheldrick, 2008 ▶); molecular graphics: *SHELXTL* (Sheldrick, 2008 ▶); software used to prepare material for publication: *SHELXTL*.

## Supplementary Material

Crystal structure: contains datablock(s) global, I. DOI: 10.1107/S160053681200596X/im2355sup1.cif

Structure factors: contains datablock(s) I. DOI: 10.1107/S160053681200596X/im2355Isup2.hkl

Supplementary material file. DOI: 10.1107/S160053681200596X/im2355Isup3.cml

Additional supplementary materials:  crystallographic information; 3D view; checkCIF report

## Figures and Tables

**Table 1:** Hydrogen-bond geometry (Å, °)

*D*—H⋯*A*	*D*—H	H⋯*A*	*D*⋯*A*	*D*—H⋯*A*
N2—H2*B*⋯N3^i^	0.86	2.25	3.057 (4)	156
N4—H4*B*⋯N1^ii^	0.86	2.24	3.027 (4)	151

Symmetry codes: (i) 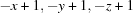; (ii) 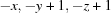.

**Fig. 1.**
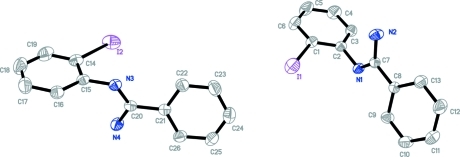
View of the two symmetry independent molecules of the title compound showing the atom numbering scheme and thermal ellipsoids at the 50% probability level.

## supplementary crystallographic information

### Comment

Benzamidines and their derivatives have attracted our attention because of their application in the synthesis of heterocyclic compounds (Attanasi *et al.* 2010, Bhosale *et al.* 2010, Deng & Mani *et al.* 20109, Wang *et al.* 2011, Ohta *et al.* 2010). We report here the crystal structure of the title compound (Fig. 1).

The asymmetric unit of the title compound contains two crystallographically independent molecules (A and B). Concerning the carbon atoms, molecule A is made up from C1 to C13 and molecule B is made up from C14 to C26. In both molecules the bond lengths and angles are generally within normal ranges. The bond lengths N1-C7 (1.293 (4) Å) and N3-C20 (1.293 (3) Å) displays double bond character, while the distances N2-C20 1.350 (5) Å and N4-C20 1.352 (5) Å are indicative of C–N single bonds. The two molecules are not parallel so that torsion angles of 8.6 (1)° between the two iodo-phenyl groups and 44.5 (2)° between both phenyl substituents, respectively, are observed. In molecule A, the planar benzene ring (C8-C13) and the iodine-substituted benzene ring (C1-C6) form a dihedral angle of 73.3 (2)°. The NH_2_ group is twisted away from the plane of the adjacent benzene ring with a dihedral angle between the N-C bond of the NH_2_ group and the plane of the adjacent phenyl ring of 18.5 (1)°. The amidine plane and both benzene rings are not coplanar showing dihedral angles of the amidine plane (N1/C7/N2) with respect to the iodine-substituted benzene ring (C1-C6) and the second benzene ring (C8-C13) of 88.2 (2)° and 51.0 (1)°, respectively. In molecule B, the planar benzene ring (C21-C26) and the iodine-substituted benzene ring (C14-C19) form a dihedral angle of 74.4 (1)°. The NH_2_ group also is twisted away from the plane of the adjacent benzene ring with a dihedral angle between the N-C bond of the NH_2_ group and the plane of the adjacent phenyl ring of 20.7 (2)°. The amidine plane in molecule B and both benzene rings are not coplanar showing dihedral angles of the amidine plane (N3/C20/N4) with respect to the iodine-substituted benzene ring (C14-C19) and the second benzene ring (C21-C26) of 95.1 (3)° and 20.7 (2)°, respectively. In the crystal structure intramolecular N—H···N (N2—H2B···N3 and N4—H4B···N1) hydrogen bonds exist (Table 1).

### Experimental

The title compound was produced according to a methodology already described in the literature (Ma *et al.* 2011, Cortes-Salva *et al.* 2011): A round bottom flask (100 mL in volume) was charged with NaH (60% in mineral oil) (360 mg, 15.0 mmol, 60%, 1.5 equiv). Under a stream of nitrogen, DMSO (10 mL) was added, and the resulting suspension was cooled with an ice-water bath prior to the addition of the 2-iodo-phenylamine (11.0 mmol, 1.1 equiv) and benzonitrile (10.0 mmol). The mixture was kept at 0 ° for 50 min and then stirred at room temperature until the starting material was consumed as monitored by TLC analysis. Ice-water (50 mL) was added while maintaining vigorous stirring. When the amidine precipitated upon addition of water, the solid was filtered off and dissolved in EtOAc (20 mL). The aqueous layer was extracted with EtOAc (3 × 20 mL). The extracts were combined and washed with water (2 × 50 mL). The organic layer was dried over Na_2_SO_4_, filtered, and concentrated under reduced pressure. The residue was purified by column chromatography (yield: 75%; m.p. 389-391 K; MS (EI, 70V): 322 (M^+^); ^1^H NMR (CDCl_3_, 400 MHz): δ = 7.88-7.86 (m, 2H), 7.47-7.05 (m, 5H), 7.47-7.05 (m, 2H), 4.86 (s, 2H)). The title compound was recrystallized from CH_2_Cl_2_ at room temperature to give the desired crystals suitable for single-crystal X-ray diffraction.

### Refinement

All H atoms were positioned geometrically and constrained to ride on their parent atoms (N—H = 0.86 Å and U_iso_(H) = 1.2U_eq_(N); C—H = 0.93 and 0.97 Å for aromatic and methylene H atoms with U_iso_(H) = 1.2U_eq_(C), respectively.

### Crystal data

**Table:**

C_13_H_11_IN_2_	*Z* = 4
*M**_r_* = 322.14	*F*(000) = 624
Triclinic, *P*1	*D*_x_ = 1.677 Mg m^−^^3^
Hall symbol: -P 1	Melting point = 389–391 K
*a* = 10.411 (3) Å	Mo *K*α radiation, λ = 0.71073 Å
*b* = 11.024 (3) Å	Cell parameters from 5918 reflections
*c* = 11.534 (3) Å	θ = 0.0–0.0°
α = 95.501 (3)°	µ = 2.49 mm^−^^1^
β = 95.065 (3)°	*T* = 296 K
γ = 102.986 (3)°	Block, colourless
*V* = 1275.7 (6) Å^3^	0.49 × 0.44 × 0.30 mm

### Data collection

**Table:**

CCD area-detector diffractometer	4716 independent reflections
Radiation source: fine-focus sealed tube	4076 reflections with *I* > 2σ(*I*)
Graphite monochromator	*R*_int_ = 0.013
φ and ω scans	θ_max_ = 25.5°, θ_min_ = 2.4°
Absorption correction: multi-scan (*SADABS*; Sheldrick, 1996)	*h* = −12→12
*T*_min_ = 0.376, *T*_max_ = 0.523	*k* = −13→13
9707 measured reflections	*l* = −13→13

### Refinement

**Table:**

Refinement on *F*^2^	Primary atom site location: structure-invariant direct methods
Least-squares matrix: full	Secondary atom site location: difference Fourier map
*R*[*F*^2^ > 2σ(*F*^2^)] = 0.033	Hydrogen site location: inferred from neighbouring sites
*wR*(*F*^2^) = 0.079	H-atom parameters constrained
*S* = 1.04	*w* = 1/[σ^2^(*F*_o_^2^) + (0.0273*P*)^2^ + 1.8412*P*] where *P* = (*F*_o_^2^ + 2*F*_c_^2^)/3
4716 reflections	(Δ/σ)_max_ = 0.001
289 parameters	Δρ_max_ = 0.94 e Å^−^^3^
0 restraints	Δρ_min_ = −1.34 e Å^−^^3^

### Special details

**Table:**

Geometry. All esds (except the esd in the dihedral angle between two l.s. planes) are estimated using the full covariance matrix. The cell esds are taken into account individually in the estimation of esds in distances, angles and torsion angles; correlations between esds in cell parameters are only used when they are defined by crystal symmetry. An approximate (isotropic) treatment of cell esds is used for estimating esds involving l.s. planes.
Refinement. Refinement of F^2^ against ALL reflections. The weighted R-factor wR and goodness of fit S are based on F^2^, conventional R-factors R are based on F, with F set to zero for negative F^2^. The threshold expression of F^2^ > 2sigma(F^2^) is used only for calculating R-factors(gt) etc. and is not relevant to the choice of reflections for refinement. R-factors based on F^2^ are statistically about twice as large as those based on F, and R- factors based on ALL data will be even larger.

### Fractional atomic coordinates and isotropic or equivalent isotropic displacement parameters (Å^2^)

**Table:**

	*x*	*y*	*z*	*U*_iso_*/*U*_eq_	
C1	0.1631 (3)	0.3372 (3)	0.1714 (3)	0.0462 (7)	
C2	0.1131 (3)	0.2143 (3)	0.1182 (3)	0.0409 (7)	
C3	0.0759 (3)	0.1972 (3)	−0.0023 (3)	0.0530 (8)	
H3	0.0421	0.1164	−0.0402	0.064*	
C4	0.0882 (5)	0.2975 (4)	−0.0663 (4)	0.0728 (11)	
H4	0.0639	0.2838	−0.1469	0.087*	
C5	0.1365 (5)	0.4185 (4)	−0.0115 (4)	0.0751 (12)	
H5	0.1441	0.4862	−0.0549	0.090*	
C6	0.1731 (4)	0.4381 (4)	0.1069 (4)	0.0629 (10)	
H6	0.2048	0.5194	0.1442	0.075*	
C7	0.1828 (3)	0.0559 (3)	0.2085 (3)	0.0390 (6)	
C8	0.1576 (3)	−0.0507 (3)	0.2811 (3)	0.0397 (6)	
C9	0.0539 (3)	−0.0646 (3)	0.3501 (3)	0.0510 (8)	
H9	0.0006	−0.0073	0.3512	0.061*	
C10	0.0289 (4)	−0.1629 (4)	0.4170 (3)	0.0618 (10)	
H10	−0.0416	−0.1715	0.4622	0.074*	
C11	0.1071 (4)	−0.2484 (4)	0.4176 (4)	0.0633 (10)	
H11	0.0905	−0.3138	0.4636	0.076*	
C12	0.2095 (4)	−0.2359 (4)	0.3499 (4)	0.0707 (11)	
H12	0.2628	−0.2933	0.3497	0.085*	
C13	0.2346 (4)	−0.1383 (4)	0.2812 (4)	0.0620 (10)	
H13	0.3039	−0.1315	0.2347	0.074*	
C14	0.4353 (3)	1.2019 (3)	0.7427 (3)	0.0515 (8)	
C15	0.4541 (3)	1.1218 (3)	0.8267 (3)	0.0405 (7)	
C16	0.5055 (3)	1.1761 (3)	0.9409 (3)	0.0529 (8)	
H16	0.5201	1.1250	0.9978	0.063*	
C17	0.5348 (4)	1.3047 (4)	0.9701 (4)	0.0737 (12)	
H17	0.5680	1.3395	1.0466	0.088*	
C18	0.5148 (5)	1.3808 (4)	0.8862 (5)	0.0827 (14)	
H18	0.5349	1.4673	0.9060	0.099*	
C19	0.4654 (4)	1.3303 (4)	0.7728 (5)	0.0722 (12)	
H19	0.4522	1.3826	0.7164	0.087*	
C20	0.3135 (3)	0.9233 (3)	0.7895 (3)	0.0398 (7)	
C21	0.2872 (3)	0.7878 (3)	0.7459 (3)	0.0418 (7)	
C22	0.3741 (4)	0.7450 (4)	0.6769 (3)	0.0574 (9)	
H22	0.4484	0.8012	0.6586	0.069*	
C23	0.3509 (4)	0.6196 (4)	0.6351 (4)	0.0738 (12)	
H23	0.4107	0.5915	0.5900	0.089*	
C24	0.2400 (4)	0.5355 (4)	0.6594 (4)	0.0672 (11)	
H24	0.2248	0.4511	0.6309	0.081*	
C25	0.1529 (4)	0.5765 (4)	0.7255 (4)	0.0629 (10)	
H25	0.0772	0.5202	0.7411	0.075*	
C26	0.1762 (4)	0.7017 (3)	0.7698 (3)	0.0550 (8)	
H26	0.1168	0.7283	0.8162	0.066*	
I1	0.22107 (3)	0.37212 (3)	0.35229 (2)	0.07680 (12)	
I2	0.36281 (3)	1.12543 (3)	0.56938 (2)	0.08108 (12)	
N1	0.0885 (2)	0.1116 (2)	0.1848 (2)	0.0423 (6)	
N2	0.3033 (3)	0.0867 (3)	0.1700 (3)	0.0553 (8)	
H2A	0.3209	0.1467	0.1271	0.066*	
H2B	0.3624	0.0464	0.1884	0.066*	
N3	0.4332 (2)	0.9910 (2)	0.7956 (2)	0.0410 (6)	
N4	0.2101 (3)	0.9688 (3)	0.8219 (3)	0.0557 (7)	
H4A	0.2222	1.0466	0.8482	0.067*	
H4B	0.1323	0.9198	0.8161	0.067*	

### Atomic displacement parameters (Å^2^)

**Table:**

	*U*^11^	*U*^22^	*U*^33^	*U*^12^	*U*^13^	*U*^23^
C1	0.0438 (17)	0.0485 (18)	0.0445 (17)	0.0066 (14)	0.0000 (14)	0.0103 (14)
C2	0.0277 (14)	0.0447 (17)	0.0514 (18)	0.0095 (12)	0.0051 (12)	0.0091 (14)
C3	0.052 (2)	0.051 (2)	0.054 (2)	0.0092 (16)	0.0055 (16)	0.0035 (16)
C4	0.087 (3)	0.081 (3)	0.046 (2)	0.011 (2)	−0.002 (2)	0.016 (2)
C5	0.099 (3)	0.062 (3)	0.062 (3)	0.008 (2)	−0.002 (2)	0.031 (2)
C6	0.073 (3)	0.045 (2)	0.064 (2)	0.0025 (18)	−0.0007 (19)	0.0133 (17)
C7	0.0287 (14)	0.0407 (16)	0.0466 (17)	0.0072 (12)	0.0035 (12)	0.0039 (13)
C8	0.0328 (15)	0.0406 (16)	0.0446 (16)	0.0085 (12)	0.0006 (12)	0.0045 (13)
C9	0.0469 (18)	0.058 (2)	0.054 (2)	0.0196 (16)	0.0124 (15)	0.0139 (16)
C10	0.061 (2)	0.073 (3)	0.059 (2)	0.0195 (19)	0.0191 (18)	0.0226 (19)
C11	0.065 (2)	0.056 (2)	0.070 (2)	0.0094 (18)	0.0055 (19)	0.0256 (19)
C12	0.067 (3)	0.059 (2)	0.099 (3)	0.029 (2)	0.018 (2)	0.029 (2)
C13	0.051 (2)	0.058 (2)	0.089 (3)	0.0241 (17)	0.025 (2)	0.026 (2)
C14	0.0382 (17)	0.061 (2)	0.061 (2)	0.0172 (15)	0.0088 (15)	0.0187 (17)
C15	0.0266 (14)	0.0473 (17)	0.0510 (18)	0.0126 (12)	0.0080 (12)	0.0098 (14)
C16	0.0467 (19)	0.057 (2)	0.056 (2)	0.0135 (16)	0.0076 (15)	0.0082 (16)
C17	0.072 (3)	0.064 (3)	0.079 (3)	0.013 (2)	0.006 (2)	−0.011 (2)
C18	0.084 (3)	0.048 (2)	0.116 (4)	0.016 (2)	0.018 (3)	0.003 (3)
C19	0.070 (3)	0.058 (2)	0.101 (4)	0.027 (2)	0.019 (2)	0.035 (2)
C20	0.0318 (15)	0.0501 (17)	0.0403 (16)	0.0131 (13)	0.0038 (12)	0.0112 (13)
C21	0.0349 (15)	0.0503 (18)	0.0403 (16)	0.0104 (13)	0.0004 (12)	0.0083 (13)
C22	0.0440 (19)	0.061 (2)	0.063 (2)	0.0069 (16)	0.0094 (16)	−0.0031 (18)
C23	0.070 (3)	0.068 (3)	0.080 (3)	0.019 (2)	0.016 (2)	−0.015 (2)
C24	0.078 (3)	0.051 (2)	0.066 (2)	0.012 (2)	−0.006 (2)	−0.0013 (18)
C25	0.065 (2)	0.052 (2)	0.068 (2)	0.0018 (18)	0.0057 (19)	0.0139 (18)
C26	0.0496 (19)	0.055 (2)	0.062 (2)	0.0105 (16)	0.0150 (16)	0.0140 (17)
I1	0.0951 (2)	0.07203 (19)	0.05011 (16)	−0.00014 (15)	−0.01240 (14)	0.00788 (12)
I2	0.0750 (2)	0.1171 (3)	0.05523 (17)	0.02581 (17)	0.00003 (13)	0.02980 (16)
N1	0.0307 (12)	0.0415 (14)	0.0573 (16)	0.0094 (10)	0.0078 (11)	0.0137 (12)
N2	0.0334 (14)	0.0629 (18)	0.079 (2)	0.0186 (13)	0.0168 (13)	0.0303 (16)
N3	0.0281 (12)	0.0465 (15)	0.0505 (15)	0.0117 (11)	0.0052 (11)	0.0094 (12)
N4	0.0317 (14)	0.0513 (16)	0.084 (2)	0.0090 (12)	0.0151 (14)	0.0037 (15)

### Geometric parameters (Å, º)

**Table:**

C1—C6	1.386 (5)	C14—I2	2.098 (4)
C1—C2	1.396 (5)	C15—C16	1.400 (5)
C1—I1	2.094 (3)	C15—N3	1.415 (4)
C2—C3	1.392 (5)	C16—C17	1.382 (5)
C2—N1	1.417 (4)	C16—H16	0.9300
C3—C4	1.376 (5)	C17—C18	1.373 (7)
C3—H3	0.9300	C17—H17	0.9300
C4—C5	1.383 (6)	C18—C19	1.378 (7)
C4—H4	0.9300	C18—H18	0.9300
C5—C6	1.368 (6)	C19—H19	0.9300
C5—H5	0.9300	C20—N3	1.293 (4)
C6—H6	0.9300	C20—N4	1.352 (4)
C7—N1	1.294 (4)	C20—C21	1.485 (4)
C7—N2	1.349 (4)	C21—C22	1.387 (5)
C7—C8	1.496 (4)	C21—C26	1.388 (5)
C8—C13	1.387 (5)	C22—C23	1.381 (6)
C8—C9	1.386 (4)	C22—H22	0.9300
C9—C10	1.381 (5)	C23—C24	1.378 (6)
C9—H9	0.9300	C23—H23	0.9300
C10—C11	1.377 (5)	C24—C25	1.360 (6)
C10—H10	0.9300	C24—H24	0.9300
C11—C12	1.367 (6)	C25—C26	1.384 (5)
C11—H11	0.9300	C25—H25	0.9300
C12—C13	1.389 (5)	C26—H26	0.9300
C12—H12	0.9300	N2—H2A	0.8600
C13—H13	0.9300	N2—H2B	0.8600
C14—C19	1.382 (6)	N4—H4A	0.8600
C14—C15	1.401 (5)	N4—H4B	0.8600
			
C6—C1—C2	121.2 (3)	C16—C15—N3	120.5 (3)
C6—C1—I1	118.7 (3)	C14—C15—N3	121.4 (3)
C2—C1—I1	120.1 (2)	C17—C16—C15	120.9 (4)
C3—C2—C1	117.4 (3)	C17—C16—H16	119.5
C3—C2—N1	120.7 (3)	C15—C16—H16	119.5
C1—C2—N1	121.7 (3)	C18—C17—C16	119.8 (4)
C4—C3—C2	121.2 (3)	C18—C17—H17	120.1
C4—C3—H3	119.4	C16—C17—H17	120.1
C2—C3—H3	119.4	C17—C18—C19	120.6 (4)
C3—C4—C5	120.4 (4)	C17—C18—H18	119.7
C3—C4—H4	119.8	C19—C18—H18	119.7
C5—C4—H4	119.8	C18—C19—C14	120.0 (4)
C6—C5—C4	119.6 (4)	C18—C19—H19	120.0
C6—C5—H5	120.2	C14—C19—H19	120.0
C4—C5—H5	120.2	N3—C20—N4	123.5 (3)
C5—C6—C1	120.2 (4)	N3—C20—C21	118.8 (3)
C5—C6—H6	119.9	N4—C20—C21	117.7 (3)
C1—C6—H6	119.9	C22—C21—C26	118.2 (3)
N1—C7—N2	123.9 (3)	C22—C21—C20	119.5 (3)
N1—C7—C8	118.7 (3)	C26—C21—C20	122.2 (3)
N2—C7—C8	117.4 (3)	C23—C22—C21	120.4 (4)
C13—C8—C9	118.1 (3)	C23—C22—H22	119.8
C13—C8—C7	121.9 (3)	C21—C22—H22	119.8
C9—C8—C7	120.0 (3)	C24—C23—C22	120.6 (4)
C10—C9—C8	120.7 (3)	C24—C23—H23	119.7
C10—C9—H9	119.7	C22—C23—H23	119.7
C8—C9—H9	119.7	C25—C24—C23	119.6 (4)
C11—C10—C9	120.7 (3)	C25—C24—H24	120.2
C11—C10—H10	119.6	C23—C24—H24	120.2
C9—C10—H10	119.6	C24—C25—C26	120.4 (4)
C12—C11—C10	119.2 (4)	C24—C25—H25	119.8
C12—C11—H11	120.4	C26—C25—H25	119.8
C10—C11—H11	120.4	C25—C26—C21	120.8 (3)
C11—C12—C13	120.5 (4)	C25—C26—H26	119.6
C11—C12—H12	119.8	C21—C26—H26	119.6
C13—C12—H12	119.8	C7—N1—C2	118.8 (2)
C8—C13—C12	120.8 (3)	C7—N2—H2A	120.0
C8—C13—H13	119.6	C7—N2—H2B	120.0
C12—C13—H13	119.6	H2A—N2—H2B	120.0
C19—C14—C15	120.7 (4)	C20—N3—C15	117.9 (2)
C19—C14—I2	119.9 (3)	C20—N4—H4A	120.0
C15—C14—I2	119.4 (3)	C20—N4—H4B	120.0
C16—C15—C14	118.0 (3)	H4A—N4—H4B	120.0
			
C6—C1—C2—C3	1.1 (5)	C14—C15—C16—C17	1.0 (5)
I1—C1—C2—C3	179.8 (2)	N3—C15—C16—C17	175.5 (3)
C6—C1—C2—N1	−172.8 (3)	C15—C16—C17—C18	−0.7 (6)
I1—C1—C2—N1	5.9 (4)	C16—C17—C18—C19	0.2 (7)
C1—C2—C3—C4	0.1 (5)	C17—C18—C19—C14	0.1 (7)
N1—C2—C3—C4	174.0 (3)	C15—C14—C19—C18	0.2 (6)
C2—C3—C4—C5	−0.9 (6)	I2—C14—C19—C18	−179.0 (3)
C3—C4—C5—C6	0.5 (7)	N3—C20—C21—C22	−22.0 (4)
C4—C5—C6—C1	0.7 (7)	N4—C20—C21—C22	158.7 (3)
C2—C1—C6—C5	−1.5 (6)	N3—C20—C21—C26	159.4 (3)
I1—C1—C6—C5	179.8 (3)	N4—C20—C21—C26	−20.0 (5)
N1—C7—C8—C13	160.1 (3)	C26—C21—C22—C23	−1.0 (5)
N2—C7—C8—C13	−19.0 (5)	C20—C21—C22—C23	−179.7 (4)
N1—C7—C8—C9	−19.3 (4)	C21—C22—C23—C24	1.2 (7)
N2—C7—C8—C9	161.7 (3)	C22—C23—C24—C25	−0.2 (7)
C13—C8—C9—C10	0.2 (5)	C23—C24—C25—C26	−1.0 (6)
C7—C8—C9—C10	179.6 (3)	C24—C25—C26—C21	1.3 (6)
C8—C9—C10—C11	0.6 (6)	C22—C21—C26—C25	−0.2 (5)
C9—C10—C11—C12	−0.8 (6)	C20—C21—C26—C25	178.4 (3)
C10—C11—C12—C13	0.0 (7)	N2—C7—N1—C2	−2.4 (5)
C9—C8—C13—C12	−1.0 (6)	C8—C7—N1—C2	178.6 (3)
C7—C8—C13—C12	179.7 (4)	C3—C2—N1—C7	96.8 (4)
C11—C12—C13—C8	0.9 (7)	C1—C2—N1—C7	−89.5 (4)
C19—C14—C15—C16	−0.7 (5)	N4—C20—N3—C15	−6.6 (5)
I2—C14—C15—C16	178.6 (2)	C21—C20—N3—C15	174.1 (3)
C19—C14—C15—N3	−175.2 (3)	C16—C15—N3—C20	101.4 (3)
I2—C14—C15—N3	4.0 (4)	C14—C15—N3—C20	−84.2 (4)

### Hydrogen-bond geometry (Å, º)

**Table:**

*D*—H···*A*	*D*—H	H···*A*	*D*···*A*	*D*—H···*A*
N2—H2*B*···N3^i^	0.86	2.25	3.057 (4)	156
N4—H4*B*···N1^ii^	0.86	2.24	3.027 (4)	151

Symmetry codes: (i) −*x*+1, −*y*+1, −*z*+1; (ii) −*x*, −*y*+1, −*z*+1.
